# ‘*He’s Practising His Learned Social Skills on the Cat*’: A Mixed-Methods Investigation of Parental Perspectives of the Role of Pets in Autistic Children’s Social Skills and Wellbeing

**DOI:** 10.3390/bs15040419

**Published:** 2025-03-25

**Authors:** Claire Wilson, Carrie Ballantyne, Roxanne D. Hawkins

**Affiliations:** 1 Division of Psychology and Social Work, School of Education and Social Sciences, University of the West of Scotland, Paisley PA1 2BE, UK; carrie.ballantyne@uws.ac.uk; 2 Department of Clinical and Health Psychology, School of Health in Social Science, University of Edinburgh, Edinburgh EH8 9AG, UK; roxanne.hawkins@ed.ac.uk

**Keywords:** pets, autism, wellbeing, parental reports

## Abstract

Evidence suggests that autistic children spend less time engaging in social interactions than their neurotypical peers which can negatively impact their wellbeing. Researchers, educators, and parents must consider how we address this. A possible facilitator of autistic children’s social skills and a protective factor for their psychological health is the role of pets and the human–pet bond. The study examined parental reports of autistic children’s attachment to their pet (dog or cat), positive and negative behaviours with that pet, and how this relates to prosocial behaviour, peer problems, and psychological health (emotional difficulties, conduct problems, hyperactivity/inattention). Sixty-five parents with an autistic child completed quantitative measures to assess these variables. Participants also completed qualitative questions aimed at understanding their perceptions of the impact of pets on their child. A regression analysis showed that children’s positive behaviour towards the pet predicted their prosocial behaviour (*β* = 0.40 *p* = 0.006). No other regression models were statistically significant. A thematic analysis of the qualitative responses highlighted four themes in relation to parents’ perceptions of the positive impacts of pets on their child. These were (1) *Anxiety, Emotion Regulation, and Sleep*; (2) *Understanding of Self and Other*; (3) *Communication, Friendships, and Social Interactions*; and (4) *Comfort and Psychological Health*. One theme was identified in relation to the negative impact of pets: (5) *Pet-Related Anxiety and Concerns*. The findings have implications which can inform guidelines to help parents make decisions about pet ownership and how to foster meaningful relationships between autistic children and their pets.

## 1. Introduction

Autism is a form of neurodivergence that affects 1 in 100 children globally (World Health Organization, 2023). Although autism is heterogeneous in nature, it commonly includes differences in social communication and interaction; repetitive and restrictive behaviours; under- or over-sensitivity to sensory information; strict adherence to routines; highly focused interests; and high anxiety and emotional problems (American Psychiatric Association, 2013). Autistic children have the desire to participate in social interactions but can face difficulties in trying to do so (Frith, 2004). This may be explained by differences in communication styles, given that autistic individuals show highly successful nuanced socio-communicative abilities when among others of a similar neurotype (Crompton et al., 2020). Thus, autistic individuals may be more socially comfortable with other autistic people rather than neurotypical people, and this can make the initiation and maintenance of social relationships with neurotypical children challenging. Autistic children often spend less time engaging in social interactions and are less likely to initiate social contact with peers (Y. Chen et al., 2021). This is problematic given that negative relationships with peers can impact upon depressive and anxiety symptoms among autistic children (Greenlee et al., 2020). Additionally, McKinlay et al. (2022) found that the perception of ‘not fitting in’ and lack of friendships between autistic children and their peers were related to poor wellbeing.

Research has shown that autistic children score higher on emotional symptoms, peer problems, conduct problems, and hyperactivity (Grasso et al., 2022; Russell et al., 2013; Salayev & Sanne, 2017) and lower on prosocial skills (Wang et al., 2022) than neurotypical children as measured via the Strengths and Difficulties Questionnaire (SDQ; Goodman, 1997). Evidence has shown that autistic children commonly report feelings of isolation, loneliness, and social anxiety (Dean et al., 2023; Fuentes et al., 2023; Lasgaard et al., 2010). This has a cyclical effect, in that social exclusion leads to symptoms of anxiety and depression, which, in turn, influences the social interactions autistic children have with their peers. Psychosocial interventions, such as Cognitive Behavioural Therapy (Hillman et al., 2020; Kreslins et al., 2015; Spain et al., 2017) and social skills training (Factor et al., 2022; Schohl et al., 2014), have shown promise in breaking this cycle. However, more research is needed to consider how to enhance social relationships and wellbeing among autistic children who may not have access to such programmes. Given that there is increasing interest in home-based interventions in improving the lives of autistic individuals (e.g., Green et al., 2010; Mullan et al., 2021) and the importance of family relationships and the home environment, a possible facilitator of social skills and protective factor for wellbeing is the role of pets and the human–pet bond.

Human–animal interactions (HAIs) research has commonly involved the use of therapy animals, showing the benefit of such animals to autistic people. In experimental settings, HAIs have had positive impacts on autistic children, such as increasing social interactions, socioemotional abilities, communication skills, conversational engagement, eye gaze, and reduced sensory sensitivities and feelings of social isolation (Ang & MacDougall, 2022; Grigore & Bazgan, 2017; London et al., 2020; O’Haire, 2013). Further, Rehn et al. (2023) examined data from seven randomised controlled trials and found that participation in animal-assisted therapy (AAT) resulted in positive trends within cognitive, social, emotional, behavioural, and physical domains for children and adolescents. Such research is promising and shows the benefits of animals to autistic children. However, it should be noted that this work has focused only on therapy animals, with relatively less work examining the role of family pets. Not every family will have the resources or access to AAT programmes, but it is more likely that families have pets. In 2024, the PDSA found that, in the UK, 51% of UK households had pets (28% owned a dog; 24% owned a cat). Findings from the AAT literature may not translate to pets, given that animals involved in AAT are commonly highly trained and the animal is present during a therapeutic intervention only, and so a strong child–animal bond may not be present. There are marked differences between a highly trained therapy animal, in a controlled and predictable environment, and a family pet (Meehan et al., 2017).

Pets are an important part of family life, and children often develop strong bonds to their companion animals, which can facilitate positive developmental outcomes for young people (Goh et al., 2023; Hawkins et al., 2023; Lisk & Lawson, 2024; Mehrotra et al., 2024). Pets can also provide physical and emotional support to humans at times of need (see Shoesmith et al., 2021). Further, N. R. Y. Chen et al.’s (2024) meta-analysis found positive relationships between pet ownership and human pro-sociality. Another positive outcome is the social-facilitating effects of pets. Pets may act as “social lubricant”, in that they provide a normalising effect and a safe topic of interest and conversation starter for autistic individuals (O’Haire, 2013). Some evidence suggests that parents report benefits of pets to their autistic child’s social skills, such as encouraging positive interactions with others and play behaviours (Carlisle, 2014; Carlisle et al., 2020). One such mechanism through which pets can facilitate socioemotional health benefits is through facilitating parent–child conversations about emotions and mental states (Reider et al., 2023). Byström and Persson (2015) argued that pets can expand social contacts, reduce anxiety, and facilitate learning among autistic children. Hawkins and Williams (2017) showed that pet attachment scores differed depending on pet type and child gender, but that an attachment to a pet can significantly increase the likelihood of increased prosocial behaviours among neurotypical children. Moreover, high-quality child–pet relationships can improve childhood developmental outcomes, including positive mental wellbeing (Kerns et al., 2023a, 2023b). However, it should be noted that research with an adult population has suggested a negative impact of pet attachment on wellbeing (e.g., Ellis et al., 2024b). Although these findings may not generalise to a child sample (for example, Scoresby et al.’s (2021) systematic review suggested a more positive impact of pets on wellbeing for children than adults), the work illustrates the importance of understanding the child–pet relationship and pet-related behaviours rather than simply ownership among autistic children. It should be noted that child–pet relationships should develop under adult supervision to ensure the animal’s needs are being met.

Pets do not automatically play an unequivocal role in every family and to every child. Having a family pet comes with increased costs and responsibilities to meet the animal’s needs. With certain pets, there is a risk of injury, such as dog bites (Schalamon et al., 2006). Children do not always engage in positive interactions with their pets (e.g., can display accidental and intentional harm); such interactions have been associated with poorer child–pet attachments and worse child outcomes, such as increased emotional and behavioural problems and poorer wellbeing (Hawkins et al., 2022, 2023; Wauthier et al., 2022). Further, such behaviours are also likely to have a negative impact on the animal’s physical and psychological wellbeing, which is problematic. It is important to examine the types of interactions that exist between autistic children and their pets due to the possible impact on both the child–pet relationship and child outcomes. Further, studies have focused predominantly on dog ownership, yet cats are the second most common pet within UK families, and emerging research has suggested that high-quality relationships may exist within child–cat dyads (Bould et al., 2018; Muldoon et al., 2019). Thus, it is important to consider children’s relationships and interactions with both dogs and cats to understand how pets may impact upon social outcomes and psychological health for autistic children.

This study examined parental reports of autistic children’s attachment to their pet (dog or cat), positive and negative behaviours with that pet, and how this relates to prosocial behaviour, peer problems, and psychological health (emotional difficulties, conduct problems, hyperactivity/inattention), controlling for sensory sensitivities, through standardised quantitative measures. We hypothesised that parental reports of child–pet attachment and positive child–pet behaviour would be positively related to prosocial behaviour and negatively related to peer problems, emotional symptoms, conduct problems, and hyperactivity. We also expected that parental reports of negative child–pet behaviour would be negatively related to prosocial behaviour and positively related to peer problems, emotional symptoms, conduct problems, and hyperactivity/inattention. This study also aimed to examine qualitative parental responses to understand lived experience of the positive and negative impacts of the child–pet relationship and child–pet interactions for autistic children. This study has significant importance in providing families with valuable insights into the potential effects of children’s relationships with family pets. The findings have implications for developing materials that will equip parents with practical guidelines on fostering meaningful and safe relationships between autistic children and their pets, which could ultimately improve child outcomes while ensuring pet welfare.

## 2. Method

### 2.1. Design and Participants

The current study employed a mixed-method, cross-sectional design using an online survey with parent-report-standardised quantitative measures and open-ended questions to collect qualitative responses. Purposive sampling was used, whereby parents with autistic children were recruited through online advertisements on social media, such as posting a flyer and link to the study on Facebook groups for parents with autistic children and groups for pet owners and advertising on X (formerly known as Twitter) with retweets from autism organisations and human–animal research groups. Parents were therefore self-selected and chose themselves whether to participate.

Following data cleaning and the removal of missing data (n = 18), the final sample included 65 parents of autistic children. This sample size was appropriate for the intended analyses based on priori power analysis using G* Power (Faul et al., 2009). Parents were aged between 31 and 53 years; the majority (n = 45) were married, were from the UK (n = 55), and were White/Caucasian (n = 58). Children were aged between 5 and 15 years (mean age = 11 years 7 months), and there were 32 girls, 32 boys, and 1 parent chose not to provide this information. Most children (n = 34) had one sibling, but some had two (n = 12) or more than two (n = 9). All but five children had a confirmed autism diagnosis. The age at diagnosis ranged from 3 years to 12 years (M = 6 years 9 months). Boys received a diagnosis at a lower average age (M = 5 years 9 months) than girls (M = 7 years 2 months). Most children (n = 40) had received some sort of support or intervention, such as school support, speech and language therapy, or art or play therapy. None of the parents mentioned animal-assisted therapy (AAT) or classroom support dogs.

Most children had an additional clinical diagnosis or difficulty as identified by parents, these included ADHD (n = 11), Tourette’s (n = 4), dyslexia (n = 4) and other learning disabilities (n = 2), anxiety (n = 9), depression (n = 2), obsessive–compulsive disorder (OCD) (n = 3), post-traumatic stress disorder (PTSD) (n = 1), sensory-processing disorders, such as Irlen syndrome, or sensitivities (n = 8), development coordination disorders, including dyspraxia (n = 5) and cerebral palsy (n = 1), fine motor skill issues (n = 1), hypermobility (n = 1), macrocephaly and demand avoidance (n = 1), and attachment issues (n = 1).

Dog ownership numbers included one dog (n = 34), two dogs (n = 10), and four dogs (n = 1). Cat ownership numbers included one (n = 21), two (n = 18), three, or more cats (n = 2). A total of 12 children had other types of pets, such as rabbits. If children had both dogs and cats or multiple pets (n = 41), they were asked to answer the survey questions based on one pet dog or cat. They were asked to “please select ONE pet to base the rest of your answers on. You may wish to choose the pet that your child is most bonded to, or spends the most time with, or has had the longest”. A total of 38 parents based their answers on a child’s pet dog, and 27 based their answers on a child’s pet cat. The age of pets ranged from 9 months to 18 years. The average age of the child when their parent acquired the pet was 6 years 7 months, with seven children being under 1 year, or the pet was in the family prior to the child’s birth. Given the possible impact on pet attachment, 36 parents reported that their child helped to choose the pet, 31 children believed the pet was their own, and 27 parents reported that their child helped to name the pet.

### 2.2. Procedure

This study was conducted according to British Psychological Society’s (BPS) ethical guidance and the Data Protection Act (2018) and was approved by the institutional ethics committee of the host university (approval number: 16542:13819). It is also important to note that the research team was experienced in collecting data from neurotypical samples and was therefore confident in data handling. The online survey was hosted on QuestionPro. At the start of the survey, parents viewed a lay summary of the project and an information sheet before deciding whether to participate. Parents then provided informed consent through an online consent form before being directed to the survey questions. The survey took approximately 20 min to complete. At the end of the survey, parents viewed a debrief form and could opt into a prize draw to win a £20 Amazon voucher.

### 2.3. Measures

*Demographics:* Basic demographics of the parent were collected, including age and gender identity, relationship status, country of residence, and race. Basic demographics of the child included child gender and age, number of siblings, autism diagnosis and any other existing clinical diagnosis, age at time of diagnosis, and whether child was/had received any intervention for their autism.

*Pet ownership:* Basic questions relating to the pet included number of pets, types of pets, pet size (dogs only), pet breed (if known), age of pets, the age of the child during pet acquisition, whether the child helped choose or name the pet, whether the child believed their pet belongs to them, whether the child sleeps with the pet at night time, the amount of time spent with the pet during weekdays and weekends, whether the pet had ever injured the child, and the reason for pet acquisition. Parents were also asked whether their child was responsible for any care-taking duties of the pet (e.g., walking, feeding, grooming, cleaning up after).

*Child behaviour toward the pet dog:* Parents were asked to report on the observed frequency of their child’s behaviour toward their pet. This measure has 29 items with 13 items relating to positive/benign behaviours and 16 items relating to negative behaviours (Arhant et al., 2017; Hawkins et al., 2022). Example items from the positive behaviour subscale are ‘speak to the pet’, ‘leave the pet alone when it is resting’, and ‘feed the pet’. The negative behaviour subscale includes items for both intentional harm (e.g., ‘inflict pain deliberately on the dog’), and unintentional harm (e.g., ‘inflict pain accidentally on the pet, e.g., stepping on’). Other example items include ‘verbally scold the pet’, ‘throw objects at the pet’, and ‘wake the pet when sleeping’. Each item is rated from 1 ‘*never*’ to 6 ‘*very often*’, and the average frequency of behaviours for each subscale was calculated with higher scores reflecting more of that behaviour. We found good internal consistency for both the positive (α = 0.82) and negative behaviour subscales (α = 0.81).

*Child–pet attachment:* The CENSHARE Pet Attachment Survey (CENSHARE-PAS; Holcomb et al., 1985) is a child self-report measure and so was adapted to be suitable for parents to report on their child’s relationship to their pet for the current study. The measure was adapted through minor wording adjustments, such as changing “you talk to your pet as a friend” to ‘your child talks to your pet as a friend’. The original measure contains 27 items rated on a 4-point Likert scale from 1 ‘*almost always*’ to 4 ‘*almost never*’. Additional items have been added in recent studies (Hawkins et al., 2022, 2023) to capture missed fundamental aspects of pet attachment, including pets as a safe-haven and pets as a safe-base (e.g., ‘your pet helps your child to enjoy exploring new places)’ and so were also included in the current study. Mean attachment scores are calculated, with higher scores indicating greater attachment. This is a widely used measure for pet attachment that is suitable for the age range tested. This measure has demonstrated strong psychometric properties and validity. We found high internal consistency of the scale (α = 0.93).

*Strengths and Difficulties:* The SDQ (Goodman, 1997) is a 25-item scale that measures a child’s internalising and externalising problems based on children’s behaviour and feelings over the past six months and is commonly used as a wellbeing indicator (Goodman et al., 2000). The measure has five subscales: emotional symptoms, conduct problems, hyperactivity, peer problems, and prosocial behaviour. Example items from the emotional symptoms scale are ‘Often unhappy, downhearted’ and ‘Many worries, often seems worried’ (α = 0.83). Example items for the conduct problems subscale include ‘Often has temper tantrums or hot tempers’ and ‘Often lies or cheats’ (α = 0.62). Hyperactivity is assessed with items such as ‘constantly fidgeting or squirming’ and ‘easily distracted, concentration wanders’ (α = 0.66). The peer problem subscale items include ‘rather solitary, tends to play alone’ and ‘has at least one good friend’ (α = 0.56). Finally, the example items from the prosocial behaviour subscale include ‘considerate of other people’s feelings’ and ‘shares readily with other children’ (α = 0.82). Each item is rated as either 0 ‘*not true*’, 1 ‘*somewhat true*’ or 2 ‘*certainly true*’ (some items are reverse scored), and total scores for each subscale were calculated.

*Sensory sensitivities:* The Parent-completed Glasgow Sensory Questionnaire (rGSQ-P; Smees et al., 2023) has 24 items that are equally distributed across seven sense domains (visual, auditory, gustatory, olfactory, tactile, vestibular, proprioception). Example items include ‘ever complain of bright lights hurting his/her eyes or causing a headache?’, ‘dislike loud noises?’, and ‘complain about the labels in clothes and ask for them to be taken out?’ Items are rated from 0 to 4: ‘never’ to ‘*always*’ (α = 0.92).

*Open-ended questions*: Qualitative questions were included for parents to provide more details about their child’s relationship and interactions with the pet. These questions focused on the perceived positive and negative impacts of the child–pet relationship. Parents were asked, ‘Do you think that your pet dog or cat has had any positive impacts on your child, particularly in relation to their autism?’ and ‘Do you feel that your pet dog or cat has had any negative impacts on your child, particularly in relation to their autism?’.

### 2.4. Data Analysis

Frequencies were calculated to explore children’s pet responsibilities, co-sleeping behaviour, and pet-related injuries. A Multiple Analysis of Variance (MANOVA) was then conducted to determine mean differences in scores for pet-positive and -negative behaviours, pet attachment, and each subscale of the SDQ as a result of dog or cat ownership. A correlational analysis was conducted to understand relationships between the study variables. Based on these correlations, hierarchical multiple regression analysis was then carried out to examine predictive validity of (1) pet-positive behaviour on children’s prosocial behaviour while controlling for the child’s age, gender, and sensory sensitivity and (2) pet attachment and positive pet behaviour on children’s emotional problems while controlling for the child’s age, gender, and sensory sensitivity. All assumptions of regression were met.

Finally, Braun and Clarke’s (2006, 2013) process of thematic analysis was used to analyse the responses to the open-text questions. This approach involves (1) familiarisation of the data through reading and re-reading the data; (2) producing initial codes; (3) searching for themes in the codes; (4) reviewing and editing themes; (5) naming and defining ‘themes’ into easily understood concepts; (6) reporting data. Themes were identified based on their relevance to the research questions and if they occurred commonly across all participants.

## 3. Results

### 3.1. Descriptive Statistics for Pet Responsibilities, Co-Sleeping, and Pet-Related Injuries

The majority of parents reported that children were responsible for some of the caregiving duties of their pet (see Table 1).

Sleeping with the pet overnight: Five parents reported that their child slept with their pet dog every night, and some reported that the dog slept on the bed most (n = 5) or some of the time (n = 11). Four parents reported that their child slept with their pet cat every night, and some reported that the cat slept on the child’s bed most (n = 5) or some (n = 12) of the time.

Pet injuries to child: Five parents reported that their pet dog had previously injured the child. Details provided highlighted that these incidents were mostly puppy bites or scratches or were accidents such as when playing. One parent mentioned their dog gave their child ‘a warning nip’, one mentioned that the dog was ‘too rough’ and ‘over excited’ with the child, and one parent mentioned that their dog had ‘snapped’ at their child. Six parents reported that their pet cat had previously injured their child. Details provided highlighted that these injuries were mostly scratches and bites; one parent reported that their cat ‘lashes out’ when over petted or handled by the child.

### 3.2. Differences in Variables According to Pet Type

The MANOVA was conducted to determine whether scores on pet attachment, pet-positive and -negative behaviour, social inclusion variables (peer problems and prosocial behaviour), and psychological health (emotional symptoms, conduct problems, hyperactivity/inattention) differed as a result of owning a pet cat or dog. Results showed that mean scores were not significantly different as a result of pet type (*F*(14, 39) = 1.56, *p* = 0.131, Wilks’ Λ = 0.64). We therefore chose not to separate the data by pet type during further analysis.

### 3.3. Descriptive Statistics and Correlations Between Pet Attachment, Child–Pet Behaviour, and Child Outcomes

Means, standard deviations, and bivariate correlation coefficients for the scales used in the study are presented in Table 2. Pet attachment was positively correlated with positive (large effect) and negative child–pet behaviours (small effect); the more attached the child was to the pet, the more likely they were to engage in both positive and negative behaviours with the pet. Pet attachment was also positively correlated with emotional problems (medium effect) and sensory sensitivities (medium effect); children with higher pet attachment were more likely to have more emotional problems and sensory sensitivity. Pet attachment was also correlated with gender (small effect). Given the way this variable was coded, this result suggests that girls had higher attachment to their pet than boys. Positive pet behaviour was positively correlated with prosocial behaviour (medium effect) and emotional problems (small effect); children who engaged in positive behaviours with their pet scored higher in prosocial behaviour but also higher in emotional problems. Negative pet behaviour was only positively correlated with sensory sensitivity (medium effect), suggesting that children with higher sensory sensitivities also exhibited more negative behaviours towards their pet. No other significant correlations were found in relation to pet attachment, pet behaviours, and internalising and externalising symptoms. It should be noted though that sensory sensitivity was positively correlated with hyperactivity (medium effect), conduct problems (medium effect), and emotional problems (medium effect). Based on these findings, regressions were carried out to further examine predictors of prosocial behaviour and emotional symptoms, given that these variables show relationships with certain pet-related variables.

### 3.4. The Predictive Role of Positive Child–Pet Behaviour on Children’s Prosocial Behaviour

Hierarchical multiple regression was used to examine the predictive role of positive child–pet behaviour on children’s prosocial behaviour while controlling for the child’s age, gender, and sensory sensitivity. Age, gender, and sensory sensitivity were added at Step 1. Pet-positive behaviour was then added at Step 2.

The model was not significant at Step 1 (*R*^2^ = 0.06 *p* = 0.415), suggesting that age, gender, and sensory sensitivity were not predictive of the child’s prosocial behaviour. Despite this, the model becomes significant at Step 2 with the inclusion of pet-positive behaviour (*R*^2^ = 0.202 *R*^2^_Change_ = 0.14 *p* = 0.006). Pet-positive behaviour was a significant predictor of prosocial behaviour (β = 0.40 *p* = 0.006). Children who engaged in more positive behaviour with their pet were more likely to demonstrate more prosocial behaviour, as reported by their parents. See Table 3.

### 3.5. The Predictive Role of Positive Child–Pet Behaviour and Pet Attachment on Children’s Emotional Problems

Hierarchical multiple regression was next used to examine the predictive role of positive child–pet behaviour and pet attachment on children’s emotional problems while controlling for the child’s age, gender, and sensory sensitivity. Age, gender, and sensory sensitivity were added at Step 1. Pet-positive behaviour and pet attachment were then added at Step 2.

The model was not significant at Step 1 (*R*^2^ = 0.12 *p* = 0.104) or Step 2 (*R*^2^ = 0.17 *p* = 0.302), suggesting that pet-positive behaviour and pet attachment were not predictive of children’s emotional problems, as reported by their parents. See Table 4.

### 3.6. Qualitative Analysis of Open-Text Questions

Thematic analysis was used to analyse the responses to the open-text questions. Four themes were identified in relation to parents’ perspectives of the positive impacts of pets on their child. These were (1) anxiety, emotion regulation and sleep; (2) understanding of self and other; (3) communication, friendships, and social interactions; and (4) comfort and psychological health. In relation to negative impacts, it should be noted that 31 participants reported that there was no negative impact of pet ownership on their child. Despite this, one additional theme was identified around negative impacts: (5) pet-related anxiety and concerns. All themes are discussed below.

#### 3.6.1. Theme 1: Anxiety, Emotion Regulation, and Sleep

It was commonly reported by parents that pet dogs and cats greatly helped children to regulate their emotions and feel calmer; this also helped to settle children at bedtime, helping them to feel ‘safe and secure’, thus improving their sleep quality. For example, children liked having pets within the bedroom at night or shortly before sleeping:

‘*Our cat helps my son to settle at night when she lies on his bed. She makes him feel safe*’

(Participant 2)

‘[Pet] *helps* [Child] *to settle at night when she is in bed. [Child] struggles to sleep but feels better when* [Pet] *is near to her*’

(Participant 25)

Dogs and cats were reported to be a calming presence throughout the day too and, importantly, helped children to regulate their emotions and reduce ‘meltdowns’, such as through physical touch and affection, and a pet’s relaxed behaviour was reflected upon the child:

‘*When my son is having a meltdown, the dog will come over and just lay with him and help him calm down*’

(Participant 59)

‘*Our dog is very relaxed when my daughter needs to cuddle her which helps her regulate and calm down*’

(Participant 65)

‘*When he is emotionally struggling or in meltdown he seeks the dog. The dog also comes over to him if he has had to be removed from a situation and calms him*’

(Participant 49)

Physical touch and proximity were also important for sensory stimulation for autistic children, offering an outlet for sensory needs:

‘[Child] *craves sensory input so stroking the cat helps him to calm down and he enjoys her kneeding and purring. He would rather sit on the floor than move her from his chair when she’s sleeping, he doesn’t want to upset her. It shows real care and affection and that he cares about her feelings. More than he would most humans! He is very protective of her*’

(Participant 44)

One caregiver mentioned that their cat provided ‘pressure therapy’, lying on a child’s body, helping them to regulate. Caregivers reported on their dog and cats’ ability to understand and respond to their child’s emotions, such as placing a paw on the child’s body when they are upset, sitting nearby, or lying next to, or close to, the child, seemingly to comfort them. Children were reported to confide in their pets when upset or worried, such as cuddling them, and were reported to talk to their pet about their worries, rather than going to a human family member. Children often accepted comfort from their pet even when they refused comfort from their caregiver.

#### 3.6.2. Theme 2: Understanding of Self and Other

Important cognitive processes seemed to be involved in child–pet relationships, such as a child learning about their own emotions and feelings through understanding their pet’s needs and feelings, for example, pets also missing caregivers when they are away, pets also needing ‘alone time’, and pets also experiencing sensory overload:

‘*We do a lot of social modelling with him. So for example our daughter has very high anxiety and leaving the house can be very difficult and being out of her safety zone. If I am away from the house our dog really misses me, and so does she, but she can comfort our dog and reassure him and in so doing is comforting herself and able to rationalize that I am coming back* via *helping out dog*’

(Participant 4)

‘*I think it helps her understand that people want and think different things*’

(Participant 15)

‘*He’s* [child] *very sensitive and understanding to the dog. And feels like they are very similar, in that the dog gets sensory overload just like him. So they almost have an understanding and he has developed a lot of patience for him*’

(Participant 44)

The above quotes demonstrate how a pet can help a child understand their own feelings. Examining parental reports highlights that pets could also aid children’s development of theory of mind, that others have their own thoughts, wants, and needs. This in turn also helped children to gain both understanding and patience. It was also highlighted that pets helped children to understand irritation and arousal, as well as unpredictability, given that pet care is not always predictable. Pets also provided children with a caregiving role and sense of responsibility and accountability:

‘*Learning to care for an animal and manage something that’s not always predictable*’

(Participant 41)

#### 3.6.3. Theme 3: Communication, Social Interactions, and Friendships

Caregivers reported that pets were helpful in aiding difficult conversations with their autistic child, being a safe and neutral being that they could ‘talk through’, therefore improving family communication:

‘*We always talk ‘through’ our dog quite often especially about things that might be hard to discuss directly*’

(Participant 4)

‘*My daughter has created an alter ego for the cat through which she finds communicating easier*’

(Participant 64)

Pets provided a comforting presence at home for the family, reducing the pressure for the child to speak in family situations. Pets facilitated ‘fun’ conversations between family members, such as creating an ‘alter ego’ for their pet and ‘giving them a voice’, which improved happiness for the whole family.

‘*Our family have created a voice/alter ego for the dog so we have pretend conversations which makes everyone laugh*’

(Participant 65)

Pets helped autistic children to communicate with others (e.g., other dog walkers), helping them practice ‘small talk’ and ‘niceties’, with pets being a safe and neutral topic of conversation. Having a shared interest empowered children to initiate interactions with others:

‘*She meets other dog walkers and talks to them, practising social small talk and niceties. Gives her confidence when out and about to acknowledge others*’

(Participant 33)

‘*The pet also gives him something to talk about and tell people about which has made some social interactions easier for him*’

(Participant 62)

Children could then practice these social skills at home when talking to their pet: ‘*Can see he’s practicing his learned social skills on the cat*’ (Participant 50). Pets also helped to improve autistic children’s confidence when communicating with others, with caregivers reporting that their child may have difficulties with conversation without the pet but has increased confidence communicating in the presence of their pet:

‘*It has helped her in social situations to have a topic to talk about and she is more confident in new situations if the dog is with us*’

(Participant 47)

Pets also helped children to interact with those who also have pets due to a shared common interest; pets therefore facilitated social interactions:

‘*Our dog has had a huge positive impact on our son. He helped to train him, and he says regularly how much our dog helps him talk to people with a common interest*’

(Participant 11)

Pets not only facilitated conversation but were viewed as sources of a friendship themselves, especially for children who were socially isolated:

‘*He* [the pet] *is her best friend, she struggles interacting with children*’

(Participant 8)

‘*The cat is her special friend, they do have a bond*’

(Participant 14)

‘*He is a constant companion even when her friends aren’t being friends*’

(Participant 17)

The stability and consistency of a pet’s friendship along with unconditional and non-judgmental aspects of a child–pet relationship were important, particularly for those who felt socially excluded and judged for being ‘different’:

‘*…the unconditional love of his dog has done wonders for him when he has felt different, excluded, and bullied. His dog will never judge him and he has an adorable relationship with her. She is his constant friend*’

(Participant 10)

#### 3.6.4. Theme 4: Comfort and Psychological Health

Pets aided psychological wellbeing through ‘brightening mood’ when a child is feeling low and through providing a sense of comfort, particularly when a child is experiencing negative emotions, being reported to be attuned and responsive to a child’s emotional cues:

‘*He instinctively goes to her when she is upset or sad and cuddling him is very comforting*’

(Participant 4)

‘*Provides comfort when he is upset. Amazingly doesn’t run off scared when he is crying but approaches and sits near. Sits on the landing outside the bedroom door like a guard dog at bedtime, even though she’s a cat*’

(Participant 56)

These accounts also reflect the strong emotional bond that exists between children and their pets. Further accounts demonstrate the strong emotional bond that can develop between a child and their pet:

‘*Our son is our dog*’*s favourite human. He plays with her better than any of the rest of the family do because he is properly invested in it. His affection towards her is utterly sincere. Because he is her favourite human she recalls to him brilliantly*’

(Participant 38)

Further, pets provide a comforting presence, providing emotional support without the child feeling pressured to communicate:

‘*The pet provides a comforting presence without the pressure to speak as my son often feels when around people*’

(Participant 62)

This is also supported by data which showed that parents reported that their child would choose to talk to and confide in their pet over another family member:

‘*When she was too sad to talk to any of us, she always would tell the dog what was wrong*’

(Participant 35)

‘*My child struggles with conversations, but not with the dog*’

(Participant 48)

‘*My son will confide how he is feeling to the dog and talk through what is happening*’

(Participant 59)

In addition, improved confidence and self-esteem was noted, for example: ‘*She* [cat] *improves self-esteem when she chooses to be with him* [son]’ (Participant 36); being ‘chosen’ by the pet over other people enhanced feelings of self-worth. In relation to mental health, it was also noted by one parent that their dog ‘*Saved her* [the child’s*] life by alerting us when she tried to commit suicide*’ (Participant 33). Thus, the pet knew when something was wrong and how to alert parents to this, demonstrating how impactful the human–pet bond can be and how attuned and responsive to human emotions dogs can be. Parents reported that pets helped children to deal better with transitions (e.g., from home to school) and to establish and maintain routines, which can promote positive wellbeing and coping ability:

‘*In the mornings, he* [cat] *comes and wakes her at the same time every day, which helps with her routine*’

(Participant 64)

‘*Walking the dog gives her a way to transition from school to home*’

(Participant 33)

‘*Leaving the house for activities that are not routine (i.e., going to school), are likely to cause* [child] *to become anxious and taking the dog reduces his anxiety and his behaviour, which is much easier on the rest of the family*’

(Participant 57)

Similarly to neurotypical studies (Shoesmith et al., 2021), parents also reported that the presence of pets increased physical activity, increased confidence in leaving the house, and increased enjoyment for participating in family walks and exploring new places; this was mostly relevant for dog owners:

‘*Child didn’t like leaving the house or physical exercise but now they have a dog she loves going for long walks*’

(Participant 47)

‘*I feel like he’s more confident and relaxed, he laughs and giggles when we are on walks and preferred them when it’s for the dog*’

(Participant 51)

‘*She also helps my child to be confident and happy to explore new places, if we didn’t have the dog, she wouldn’t go*’

(Participant 61)

#### 3.6.5. Theme 5: Pet-Related Anxieties and Concerns

This theme concerns parents’ reports that, in certain contexts, their child worries and has anxieties about their pet and relates to parental concerns regarding child–pet interactions. For example, it appeared common for children to have separation anxiety when away from home and not with their pet:

‘*The only concern is that she* [child] *is so attached to him* [pet] *that going on holiday without him is really hard but we have managed that situation with lots of preparation and having him looked after by close friends who can keep us in contact with how he’s doing*’

(Participant 4)

‘*She misses her a lot when away from home*’

(Participant 25)

‘*Sometimes she worries about the dog if they are separated*’

(Participant 19)

Negative emotions were often expressed in the absence of a pet, which may be explained by the importance of the pet for the child’s emotional regulation, comfort, and companionship as described above. A child’s worries and anxieties were often in relation to a pet’s health, safety, and wellbeing, and such concerns negatively impacted upon a child’s wellbeing:

‘*Makes him anxious if the cat isn’t in the house when he goes to bed*’

(Participant 7)

‘*If she* [pet] *hurts herself, he* [child] *can really worry*’

(Participant 10)

‘*I do worry about when pet dies how this will affect son and how he will cope as they are so close*’

(Participant 30)

‘*Once the cat went missing and my child was beyond distraught. We were really concerned for their* [child’s] *wellbeing*’

(Participant 63)

Parents also expressed concerns regarding child–pet interactions and reported that pet care was sometimes ‘too much’ for the child.

‘*He loves dog like a brother and worries for him. He struggles with remembering to play with him, but they are always close to each other either*’

(Participant 30)

‘*The responsibility of having to care for her and walk her is too much sometimes*’

(Participant 42)

Pets could sometimes negatively impact a child due to sensory sensitivity:

‘*The new puppy is quite barky so as our son has noise sensitivity with his ASD he finds the barking difficult*’

(Participant 20)

‘*Sometimes if they* [Pet] *are dirty or smell she* [Child] *doesn’t like it*’

(Participant 15)

Although sensory stimulation from a pet was often perceived as positive, this was also described by parents as sometimes ‘excessive’, impacting upon a pet’s welfare, along with interactions that were too ‘rough’ that could harm the animal:

‘*Tactile seeking made it very difficult in the first few months as my daughter found it hard to leave the kittens alone*’

(Participant 26)

‘*It has been hard for my daughter at times to understand that the dog doesn’t always want attention. At the beginning she used to get very upset about it. Especially on the occasions he has given her a warning growl, it has triggered meltdowns*’

(Participant 47)

## 4. Discussion

This study investigated parental reports of autistic children’s attachment to their pet (dog or cat), positive and negative behaviours with that pet, and how this relates to prosocial behaviour, peer problems, and psychological health (emotional difficulties, conduct problems, hyperactivity/inattention). Specifically, we hypothesised that parental reports of child–pet attachment and positive child–pet behaviour would be positively related to prosocial behaviour and negatively related to peer problems, emotional symptoms, conduct problems, and hyperactivity. The first hypothesis was partially upheld. We found that children who engaged in more positive behaviours with their pet were more likely to demonstrate more prosocial behaviour. However, we did not find that child–pet attachment and positive child–pet behaviour were related to aspects of psychological health. No other hypotheses were supported. This study also examined qualitative parental responses to understand lived experience of the positive and negative impacts of the child–pet relationship and child–pet interactions for autistic children. Five themes were identified, which help to better situate the quantitative results.

Our findings demonstrated a positive association between positive pet behaviour and prosocial behaviour among autistic children, as reported by their parents. This supports and extends previous works, which found a relationship between pet ownership and prosocial behaviour among neurotypical children (N. R. Y. Chen et al., 2024; Christian et al., 2020). Our study suggests that in addition to pet ownership, it is important to consider the types of behaviour that the child engages in with the pet (e.g., positive vs. negative behaviours). Our qualitative findings further support this assertation by showing that engaging in caring behaviours with the pet (under parents’ supervision) can give the child a sense of responsibility and accountability. In addition, we found that pets can help autistic children understand the needs, thoughts, and feelings of others. This may provide an explanation for the quantitative relationship between pet-positive behaviour and prosocial behaviour, in that pets help autistic children act in line with others’ needs. Research has suggested that autistic individuals can find it difficult to recognise others’ mental states (i.e., cognitive empathy; Fatima & Babu, 2024; Kimmig et al., 2024; Shalev et al., 2022; Sönmez & Jordan, 2024; Vilas et al., 2021), and, thus, caring for a pet may allow children to practice this skill. Future research is needed which examines the relationship between autistic children’s pet behaviour, cognitive empathy, and prosocial behaviour.

Interestingly, parents’ reports of their child’s attachment to the pet were not related to prosocial behaviour. Previous research has found links between neurotypical children’s pet attachment and prosocial behaviour (Hawkins & Williams, 2017; Li, 2022), suggesting that the impact of pet attachment upon prosocial behaviour may be different for autistic children. More research is needed to further examine the nature of autistic children’s pet attachments and how this relates to behaviour.

Our quantitative findings also indicated no significant relationships between pet attachment/behaviours and the child’s peer problems. Thus, parents’ reports of the child’s pet bond or pet-related behaviours did not impact upon reports of their relationships with peers. In contrast, Kerns et al. (2017) found that for neurotypical children, being closer to their pet was related to higher quality relationships with peers and less conflict. Our finding is interesting when considered in the context of the qualitative responses. The *Communication, Social Interactions, and Friendship* theme suggested that parents believed that pets enhanced interaction opportunities for their child. This supports previous research which has argued that pets can act as a ‘social lubricant’ for autistic children and encourage positive interactions with others (Carlisle, 2014; Carlisle et al., 2020; O’Haire, 2013). However, having a positive social interaction does not automatically create a friendship between individuals (Carrier, 1999). Thus, although autistic children may experience more interactions because of the pet, it cannot be assumed that this will correlate with friendships or lack of peer problems.

It should be noted though that within the *Communication, Social Interactions, and Friendship* theme was the acknowledgement that pets were seen as an alternative to friends. Some children were reported to trust the pet more than humans and viewed the pet as a friend who had unconditional love for them. Animals can be viewed as easier to create bonds with, given their non-judgemental nature, availability, and unconditional support. Thus, some children may form such a close bond with the pet that their social needs are saturated and therefore feel less of a desire to bond with humans. Although pets may not address the child’s peer problems, pets can provide the child with a different kind of companionship. Indeed, within the *Comfort and Psychological Health* theme, it was noted that children often chose to confide in the pet over a human and liked their pet’s presence without the pressure to speak. This supports research with autistic adults that has found animals can provide an alternative way to experience social interaction that was free from the stressors involved in human contact (Atherton et al., 2023). It also supports O’Haire et al.’s (2015) hypothesis that pets act as a social buffer against the effects of stress.

In relation to psychological health, our quantitative results showed no relationships between parents’ reports of their child’s pet attachment/behaviour and emotional difficulties. Some previous research has found positive relationships between these variables in both autism and neurotypical samples (Byström & Persson, 2015; Kerns et al., 2023a, 2023b). It should be noted though that Grandgeorge et al. (2012) found that the benefit of pet ownership to emotional difficulties came in the initial stages of having the pet in an autistic population. Therefore, it seems if the pet was in the household for many years, the same benefit may not be found, as compared to those who recently acquired a pet. It is also unclear how long these benefits remained after the pets’ arrival. This is further supported by a recent study which found that the benefits of pet ownership for wellbeing may be more likely within the initial stages of ownership among a neurotypical sample (Ellis et al., 2024a). In our study, the average age of the children was 11 years and 7 months, whereas parents reported the average age of the child when acquiring the pet was 6 years and 7 months. Thus, the lack of relationship between pet variables and emotional problems may be a result of length of time the family have had the pet. Future studies could therefore examine pet ownership duration and wellbeing benefits within the autistic population.

Despite this, the qualitative findings suggest a contradictory argument. For example, the *Anxiety, Emotion Regulation, and Sleep* theme represented parental beliefs that pet dogs and cats help their children’s emotional regulation, feelings of calmness, reduced ‘meltdowns’, and helped sleep. This aligns with research recruiting neurotypical children (Yu, 2024). Our finding is important, given evidence that has shown that autistic children have difficulties with emotional self-regulations, such as emotion management, response inhibition, and delaying gratification (American Psychiatric Association, 2013; Baron et al., 2006; Laurent & Gorman, 2018). Further, Konstantareas and Stewart’s (2006) work has demonstrated that autistic children tend to show less frequent use of effective emotion-regulation strategies than neurotypical children. Through the lens of emotion-regulation theories, pets can help children manage their emotions and understand irritation, arousal, and others’ thoughts. Our *Comfort and Psychological health* theme also suggested that pets aided psychological wellbeing through ‘brightening mood’ when a child was feeling low and through providing a sense of comfort. In addition to this, parents also stated that pets can enhance children’s confidence, self-esteem, and physical activity. A key mechanism commonly reported in the qualitative data here was the important role of physical touch and affection from pets, which aligns with previous work (e.g., Young et al., 2020). Further, our findings support that of Hawkins et al. (2024), who found that pets are attuned to neurotypical children’s emotional cues. One parent within our sample explained how the pet dog had alerted them to their child’s suicide attempt. Related to this and in support of previous research (e.g., Reider et al., 2023) is our finding within the *Communication, Interaction, and Friendship* theme that pets were helpful in aiding difficult conversations with their autistic child, being a safe and neutral being that they could ‘talk through’.

What should be noted though is that the *Pet-related Anxieties and Concerns* theme reported on parental concerns that their pet may cause their child to experience separation anxiety and worry around the pet’s health and safety. Further, although some parents reported a positive impact of sensory stimulation from a pet, others believed this could have a negative impact upon their child due their sensory sensitivity. Further, other parents noted that at times when the pet did not want to play or want affection, this could cause distress for their child. This suggests a complex relationship between autistic children’s pet relationship and wellbeing/emotional problems, whereby the pet can reduce some anxieties while increasing other types of anxiety. Such nuances may have been missed by the quantitative measures used in this study. Future research should take this into account when considering the impact of pets on autistic children’s wellbeing. Research also needs to consider this from a pet welfare perspective. For example, insights on how parents ensure both the child’s and the pet’s need are met is needed.

Our study found no relationships between pet variables and conduct problems or hyperactivity. Although Christian et al. (2020) found that pet ownership was associated with poorer outcomes relating to neurotypical children’s attention span and ability to complete tasks, there is limited research focusing on this. Given that our qualitative findings suggested that pets enhance autistic children’s feelings of calmness and comfort, it is surprising that this has not been shown in these quantitative measures and is thus an area for future research.

We also found that none of the psychological wellbeing variables were associated with negative behaviours towards the pet. Further, descriptive data demonstrated that the mean score for negative pet behaviours was much lower than pet-positive behaviours. Thus, parents were more likely to report that their child engaged in caring behaviours rather than harmful behaviours (intentional or unintentional) towards their pet. From an animal welfare perspective, this is a positive finding, which suggests that autistic children are more engaged in positive than negative behaviours with their pet. However, it should be noted that one participant qualitatively reported that their dog would give the child a ‘warning growl’ when they needed space. This was the exception though as no other parent qualitatively reported child behaviours which suggested the child caused harm to the pet. Such a finding should be considered in line with a previous work, which suggested that children and animals can have a great impact upon each other’s lives (Hawkins & Williams, 2017). Future research is needed which also focuses on pets’ wellbeing.

## 5. Implications

This study has significant importance in providing families with valuable insights into the potential effects of autistic children’s relationships with family pets. The findings have implications for developing practical guidelines which will help parents make the decision as to whether to bring a pet into the family as well as how to foster meaningful and safe relationships between the pet and their autistic child. For example, parents should allow and support their child to take on a caring role with the pet to enhance children’s feelings of responsibility and accountability. This may have a positive impact upon their child’s understanding of others and on their prosocial behaviour. Further, it is important that parents understand the impact of sensory stimulation. This will be beneficial to some autistic children’s wellbeing while being less important or perhaps detrimental to others. The pet’s wellbeing must also be closely monitored during child–pet interactions to support pet welfare and to reduce risk of harm. Parents must also understand the impact of pet health and separation anxiety on the child’s wellbeing. Pets may enhance the child’s confidence, physical activity, and social interactions, but parents should not automatically assume this will translate into increased friendships for their child. Despite this, our findings suggest that a family pet can foster communication about difficult topics with the child and family members. Such recommendations will allow families with an autistic child to enjoy a pet while having a positive impact on child outcomes.

## 6. Limitations

Possible limitations of the study should be acknowledged. Self-report questionnaires may increase the likelihood of measurement bias and socially desirable responding (Campbell & Fiske, 1959). However, Podsakoff et al.’s (2003) procedural remedies were used to minimise this risk. Participants used the full range of response options, which increased our confidence in the validity of the results. Although there are benefits to online research, it must be acknowledged that there was potential for self-selection bias (Bethlehem, 2010). The findings should be considered in light of this. Another limitation relates to our measure of psychological health. Firstly, some of the SDQ sub-scales showed low internal consistency (e.g., peer problems α = 0.56, conduct problems α = 0.62). In addition, our qualitative findings suggested the need to use a measure which may consider different types of child anxieties. This is an area for future research. Related to this point was the cross-sectional nature of our study, and thus, we were not able to track the impact of pets and pet attachment over time. Our findings suggest the need for longitudinal research which examines how the child–pet bond and behaviours are related to child outcomes over time. Further, despite the favourable notion of investigating parental reports, some research has suggested parent and child viewpoints may differ when asked about specific topics (Hou et al., 2020). It would be useful for future research to also include child reports and behavioural observations. It would also be useful for future research to consider the socio-economic status of families involved, given that this may impact pet ownership (Purewal et al., 2019). Finally, it should be noted that that many parents reported that their child had additional diagnoses, such as ADHD, dyslexia, dyspraxia, OCD, and PTSD. Given our sample size, we were not able to control for co-morbidities in the analysis. Thus, the observed effects may be driven by other factors that we did not measure.

## 7. Conclusions

To conclude, our quantitative data showed a positive association between positive pet behaviour and prosocial behaviour among autistic children. The qualitative results echoed this, showing that pets can help autistic children understand the needs of others (both animals and humans), and caring for a pet may therefore allow children to practice cognitive empathy skills. Although pet attachment and behaviours were not statistically related to children’s peer problems, qualitatively, we found that pets enhanced human interaction opportunities for their child. We also found that pets may provide an alternative way to experience social interaction that was free from the stressors involved in human contact. In relation to psychological health, our quantitative results showed no relationships between parents’ reports of their child’s pet attachment/behaviour and emotional difficulties. Despite this, the qualitative findings suggested that pets helped children’s emotional regulation, feelings of calmness and comfort, reduced ‘meltdowns’, and helped sleep, with a key mechanism underpinning these effects being the attunement of pets to children’s emotional cues. However, it should be acknowledged that pets may heighten children’s anxieties around separating from the pet and the pet’s health and safety. Future research may wish to further consider species-specific differences in attunement. No relationships between pet variables and conduct problems or hyperactivity were found. The findings have implications for developing practical guidelines to help parents make the decisions around pet ownership as well as how to foster meaningful and safe relationships between the pet and their autistic child.

## Figures and Tables

**Table 1:** Frequencies for children’s pet responsibilities split by pet type.

Responsibilities	Walking	Feeding	Grooming	Clean Up After	All Duties	Most Duties	Some Duties	None
Dog owners (n = 38)	14	13	9	7	0	4	28	6
Cat owners (n = 27)	n/a	9	2	1	0	1	17	9

**Table 2:** Mean (and standard deviation) scores for measured variables.

	Attch	Pet_p	Pet_n	Proso	Peer	Hyper	Emot	Cond	Sens	Age	Gend	Mean	SD
Attch		0.77 ***	0.27 *	0.17	0.13	−0.07	0.31 *	0.17	0.38 **	0.03	−0.27 *	123.77	28.42
Pet_p			0.39 **	0.31 *	0.11	0.03	0.29 *	0.12	0.24	−0.08	−0.22	53.60	11.42
Pet_n				−0.01	0.17	0.09	0.03	0.14	0.37 **	−0.18	−0.24	27.95	9.13
Proso					0.04	−0.34 *	0.06	−0.20	−0.13	−0.15	0.12	10.48	2.70
Peer						−0.11	0.20	0.07	0.16	−0.01	−0.14	10.42	1.55
Hyper							0.00	0.33 *	0.31 *	0.10	−0.20	11.76	2.30
Emot								0.10	0.31 *	−0.09	−0.19	11.31	1.97
Cond									0.46 ***	0.14	0.16	8.19	1.47
Sens										−0.07	−0.18	67.78	12.54
Age											0.19	11.79	7.75
Gend													

* *p* < 0.05; ** *p* < 0.01; *** *p* < 0.001; Age = child’s age; Gend = child’s gender; Attch = pet attachment; Pet_p = pet-positive behaviour; Pet_n = pet-negative behaviour; Proso = prosocial behaviour; Peer = peer problems; Hyper = hyperactivity; Emot = emotional problems; Cond = conduct problems; Sens = sensory sensitivity.

**Table 3:** Pet-positive behaviour as a predictor of prosocial behaviour.

Step and Predictors	*R* ^2^	*R* ^2^ *_Change_*	*F_Change_*	β	*p*
**Step 1**	0.06	0.06	0.97		
Age				−0.20	0.187
Gender				0.16	0.277
Sensory				−0.07	0.611
**Step 2**	0.20	0.14	8.30 **		
Age				−0.19	0.170
Gender				0.24	0.096
Sensory				−0.15	0.287
Pet_P				0.40 **	0.006

** *p* < 0.01; Pet_P = pet-positive behaviour.

**Table 4:** Pet-positive behaviour and attachment as predictors of emotional problems.

Step and Predictors	*R* ^2^	*R* ^2^ *_Change_*	*F_Change_*	β	*p*
**Step 1**	0.12	0.12	2.17		
Age				−0.04	0.781
Gender				−0.13	0.359
Sensory				0.29	0.045
**Step 2**	0.17	0.05	1.23		
Age				−0.05	0.720
Gender				−0.09	0.547
Sensory				0.22	0.151
Pet_P				0.10	0.662
Pet_Attch				0.15	0.516

Pet_P = pet-positive behaviour; Pet_Attch = pet attachment.

## Data Availability

The datasets presented in this article are not readily available because of the level of consent obtained from participants.

## References

[B1-behavsci-15-00419] American Psychiatric Association (2013). Diagnostic and statistical manual of mental disorders *(DSM-5^®^)*.

[B2-behavsci-15-00419] Ang C. S., MacDougall F. A. (2022). An evaluation of animal-assisted therapy for Autism Spectrum Disorders: Therapist and parent perspectives. Psychological Studies.

[B3-behavsci-15-00419] Arhant C., Beetz A., Troxler J. (2017). Caregiver reports of interactions between children up to 6 years and their family dog—Implications for dog bite prevention. Frontiers in Veterinary Science.

[B4-behavsci-15-00419] Atherton G., Edisbury E., Piovesan A., Cross L. (2023). ‘They ask no questions and pass no criticism’: A mixed-methods study exploring pet ownership in autism. Journal of Autism and Developmental Disorders.

[B5-behavsci-15-00419] Baron G., Groden J., Groden G., Lipsitt L. P. (2006). Stress and coping in autism.

[B6-behavsci-15-00419] Bethlehem J. (2010). Selection bias in web surveys. International Statistical Review.

[B7-behavsci-15-00419] Bould E., Bigby C., Bennett P., Howell T. J. (2018). ‘More people talk to you when you have a dog’—Dogs as catalysts for social inclusion of people with intellectual disabilities. Journal of Intellectual Disability Research.

[B8-behavsci-15-00419] Braun V., Clarke V. (2006). Using thematic analysis in psychology. Qualitative Research in Psychology.

[B9-behavsci-15-00419] Braun V., Clarke V. (2013). Successful qualitative research: A practical guide for beginners.

[B10-behavsci-15-00419] Byström K. M., Persson C. A. L. (2015). The meaning of companion animals for children and adolescents with autism: The parents’ perspective. Anthrozoös.

[B11-behavsci-15-00419] Campbell D. R., Fiske D. W. (1959). Convergent and discriminant validation by the multitrait-multimethod matrix. Psychological Bulletin.

[B12-behavsci-15-00419] Carlisle G. K. (2014). Pet dog ownership decisions for parents of children with autism spectrum disorder. Journal of Pediatric Nursing.

[B13-behavsci-15-00419] Carlisle G. K., Johnson R., Wang Z., Brosi T., Rife E., Hutchison A. (2020). Exploring human–companion animal interaction in families of children with Autism. Journal of Autism and Developmental Disorders.

[B14-behavsci-15-00419] Carrier J. G. (1999). People who can be friends: Selves and social relationships.

[B15-behavsci-15-00419] Chen N. R. Y., Majeed N. M., Lai G. J., Koh P. S., Kasturiratna S., Manmeet Kaur A. Z. Y., Yong H. J. C., Hartanto A. (2024). Human–animal interaction and human prosociality: A meta-analytic review of experimental and correlational studies. Anthrozoös.

[B16-behavsci-15-00419] Chen Y., Senande L., Thorsen M., Patten K. (2021). Peer preferences and characteristics of same-group and cross-group social interactions among autistic and non-autistic adolescents. Autism.

[B17-behavsci-15-00419] Christian H., Mitrou F., Cunneen R., Zubrick S. R. (2020). Pets are associated with fewer peer problems and emotional symptoms, and better prosocial behavior: Findings from the longitudinal study of Australian children. The Journal of Pediatrics.

[B18-behavsci-15-00419] Crompton C., Ropar D., Evans-Williams C., Flynn E., Fletcher-Watson S. (2020). Autistic peer-to-peer information transfer is highly effective. Autism.

[B19-behavsci-15-00419] Data Protection Act (2018). https://www.legislation.gov.uk/ukpga/2018/12/contents/enacted.

[B20-behavsci-15-00419] Dean M., Chang Y. C., Shih W., Orlich F., Kasari C. (2023). Social engagement and loneliness in school-age autistic girls and boys. Women’s Health.

[B21-behavsci-15-00419] Ellis A., Loughnan S., Hawkins R. D., Stanton S. C. (2024a). The associations between human–companion animal relationship duration, companion animal life stage, and relationship quality. Animals.

[B22-behavsci-15-00419] Ellis A., Stanton S. C. E., Hawkins R. D., Loughnan S. (2024b). The link between the nature of the human–companion animal relationship and well-being outcomes in companion animal owners. Animals.

[B23-behavsci-15-00419] Factor R. S., Moody C. T., Sung K. Y., Laugeson E. A. (2022). Improving social anxiety and social responsiveness in autism spectrum disorder through PEERS^®^. Evidence-Based Practice in Child and Adolescent Mental Health.

[B24-behavsci-15-00419] Fatima M., Babu N. (2024). Cognitive and affective empathy in autism spectrum disorders: A meta-analysis. Review Journal of Autism and Developmental Disorders.

[B25-behavsci-15-00419] Faul F., Erdfelder E., Buchner A., Lang A. G. (2009). Statistical power analyses using G*Power 3.1: Tests for correlation and regression analyses. Behavior Research Methods.

[B26-behavsci-15-00419] Frith U. (2004). Emanuel Miller lecture: Confusions and controversies about Asperger syndrome. Journal of Child Psychology and Psychiatry and Allied Disciplines.

[B27-behavsci-15-00419] Fuentes C., Flores-Vergara S., Gálvez-Zurita J., Urra-Albornoz C., la Torre Choque C., Bolaños M. C. (2023). Comparison of social isolation in autistic children and adolescents according to age, marital status and number of siblings. Journal of Education and Health Promotion.

[B28-behavsci-15-00419] Goh A. Y. H., Chia S. M., Majeed N. M., Chen N. R. Y., Hartanto A. (2023). Untangling the additive and multiplicative relations between natural scenery exposure and human–animal interaction on affective well-being: Evidence from daily diary studies. Sustainability.

[B29-behavsci-15-00419] Goodman R. (1997). The strengths and difficulties questionnaire: A research note. Journal of Child Psychology and Psychiatry.

[B30-behavsci-15-00419] Goodman R., Ford T., Simmons H., Gatward R., Meltzer H. (2000). Using the strengths and difficulties questionnaire (SDQ) to screen for child psychiatric disorders in a community sample. British Journal of Psychiatry.

[B31-behavsci-15-00419] Grandgeorge M., Tordjman S., Lazartigues A., Lemonnier E., Deleau M., Hausberger M. (2012). Does pet arrival trigger prosocial behaviors in individuals with autism?. PLoS ONE.

[B32-behavsci-15-00419] Grasso M., Lazzaro G., Demaria F., Menghini D., Vicari S. (2022). The strengths and difficulties questionnaire as a valuable screening tool for identifying core symptoms and behavioural and emotional problems in children with neuropsychiatric disorders. International Journal of Environmental Research and Public Health.

[B33-behavsci-15-00419] Green J., Charman T., McConachie H., Aldred C., Slonims V., Howlin P., Le Couteur A., Leadbitter K., Hudry K., Byford S., Barrett B., Temple K., Macdonald W., Pickles A., PACT Consortium (2010). Parent-mediated communication-focused treatment in children with autism (PACT): A randomised controlled trial. Lancet.

[B34-behavsci-15-00419] Greenlee J., Winter M., Johnson M. (2020). Depression symptoms in adolescents with autism spectrum disorder: A contextual approach to mental health comorbidities. Journal of Adolescence.

[B35-behavsci-15-00419] Grigore A. N., Bazgan M. (2017). Effects of assisted animal therapy on the development of socio-emotional abilities of children with autism. Bulletin of the Transilvania University of Brasov. Series VII, Social Sciences and Law.

[B36-behavsci-15-00419] Hawkins R. D., Kuo C. H., Robinson C. (2024). Young adults’ views on the mechanisms underpinning the impact of pets on symptoms of anxiety and depression. Frontiers in Psychiatry.

[B37-behavsci-15-00419] Hawkins R. D., Robinson C., Brodie Z. P. (2022). Child–dog attachment, emotion regulation and psychopathology: The mediating role of positive and negative behaviours. Behavioral Sciences.

[B38-behavsci-15-00419] Hawkins R. D., Robinson C., McGuigan N. (2023). The benefits and risks of child-dog attachment and child-dog behaviours for child psychological well-being. Human-Animal Interactions.

[B39-behavsci-15-00419] Hawkins R. D., Williams J. (2017). Childhood attachment to pets: Associations between pet attachment, attitudes to animals, compassion, and humane behaviour. International Journal of Environmental Research and Public Health.

[B40-behavsci-15-00419] Hillman K., Dix K., Ahmed K., Lietz P., Trevitt J., O’Grady E., Uljarević M., Vivanti G., Hedley D. (2020). Interventions for anxiety in mainstream school-aged children with autism spectrum disorder: A systematic review. Campbell Systematic Reviews.

[B41-behavsci-15-00419] Holcomb R., Williams R. C., Richards P. S. (1985). The elements of attachment: Relationship maintenance and intimacy. Journal of the Delta Society.

[B42-behavsci-15-00419] Hou Y., Benner A. D., Kim S. Y., Chen S., Spitz S., Shi Y., Beretvas T. (2020). Discordance in parents’ and adolescents’ reports of parenting: A meta-analysis and qualitative review. American Psychologist.

[B43-behavsci-15-00419] Kerns K. A., Dulmen M. H., Kochendorfer L. B., Gastelle M., Obeldobel C. A., Horowitz A. (2023a). Are children’s relationships with pet dogs uniquely related to children’s social and emotional competence and adjustment?. Human-Animal Interactions.

[B44-behavsci-15-00419] Kerns K. A., Koehn A. J., van Dulmen M. H. M., Stuart-Parrigon K. L., Coifman K. G. (2017). Preadolescents’ relationships with pet dogs: Relationship continuity and associations with adjustment. Applied Developmental Science.

[B45-behavsci-15-00419] Kerns K. A., Obeldobel C. A., House H., Kochendorfer L. B., White A., Gastelle M. (2023b). Children’s experiences of positive affect with pet dogs: A multi-method study. Human-Animal Interactions.

[B46-behavsci-15-00419] Kimmig A. C. S., Burger L., Schall M., Derntl B., Wildgruber D. (2024). Impairment of affective and cognitive empathy in high functioning autism is mediated by alterations in emotional reactivity. Scientific Reports.

[B47-behavsci-15-00419] Konstantareas M. M., Stewart K. (2006). Affect regulation and temperament in children with Autism Spectrum Disorder. Journal of Autism and Developmental Disorders.

[B48-behavsci-15-00419] Kreslins A., Robertson A. E., Melville C. (2015). The effectiveness of psychosocial interventions for anxiety in children and adolescents with autism spectrum disorder: A systematic review and meta-analysis. Child Adolescent Psychiatry Mental Health.

[B49-behavsci-15-00419] Lasgaard M., Nielsen A., Eriksen M. E., Goossens L. (2010). Loneliness and social support in adolescent boys with autism spectrum disorders. Journal of Autism and Developmental Disorders.

[B50-behavsci-15-00419] Laurent A. C., Gorman K. (2018). Development of emotion self-regulation among young children with autism spectrum disorders: The role of parents. Journal of Autism and Developmental Disorders.

[B51-behavsci-15-00419] Li J. (2022). The research on the correlation among pet attachment, prosociality, social support and loneliness based on MF-DCCA. Academic Journal of Computing & Information Science.

[B52-behavsci-15-00419] Lisk C., Lawson L. M. (2024). Investigating human-animal interactions in homes of children with Autism Spectrum Disorders: A mixed-methods approach. Society & Animals.

[B53-behavsci-15-00419] London M. D., Mackenzie L., Lovarini M., Dickson C., Alvarez-Campos A. (2020). Animal assisted therapy for children and adolescents with autism spectrum disorder: Parent perspectives. Journal of Autism and Developmental Disorders.

[B54-behavsci-15-00419] McKinlay J., Wilson C., Hendry G., Ballantyne C. (2022). “It feels like sending your children into the lions’ den”—A qualitative investigation into parental attitudes towards ASD inclusion, and the impact of mainstream education on their child. Research in Developmental Disabilities.

[B55-behavsci-15-00419] Meehan M., Massavelli B., Pachana N. (2017). Using attachment theory and social support theory to examine and measure pets as sources of social support and attachment figures. Anthrozoös.

[B56-behavsci-15-00419] Mehrotra M., White N., Foley S., Hughes C. (2024). Sibling and pet relationships: Links with adversity and adjustment in pre-adolescence. Applied Developmental Science.

[B57-behavsci-15-00419] Muldoon J. C., Williams J. M., Lawrence A., Currie C. (2019). The nature and psychological impact of child/adolescent attachment to dogs compared with other companion animals. Society & Animals.

[B58-behavsci-15-00419] Mullan A., Boyd K., McConkey R. (2021). The impact of a brief home-based intervention on families with a child with Autism Spectrum Disorder. Journal of Developmental and Physical Disabilities.

[B59-behavsci-15-00419] O’Haire M. E. (2013). Animal-assisted intervention for autism spectrum disorder: A systematic literature review. Journal of Autism and Developmental Disorders.

[B60-behavsci-15-00419] O’Haire M. E., McKenzie S. J., Beck A. M., Slaughter V. (2015). Animals may act as social buffers: Skin conductance arousal in children with autism spectrum disorder in a social context. Developmental Psychobiology.

[B61-behavsci-15-00419] Podsakoff P. M., MacKenzie S. B., Lee J. Y., Podsakoff N. P. (2003). Common method biases in behavioral research: A critical review of the literature and recommended remedies. Journal of Applied Psychology.

[B62-behavsci-15-00419] Purewal R., Christley R., Kordas K., Joinson C., Meints K., Gee N., Westgarth C. (2019). Socio-demographic factors associated with pet ownership amongst adolescents from a UK birth cohort. BMC Veterinary Research.

[B63-behavsci-15-00419] Rehn A. K., Caruso V. R., Kumar S. (2023). The effectiveness of animal-assisted therapy for children and adolescents with autism spectrum disorder: A systematic review. Complementary Therapies in Clinical Practice.

[B64-behavsci-15-00419] Reider L. B., Kim E., Mahaffey E., LoBue V. (2023). The impact of household pets on children’s daily lives: Differences in parent–child conversations and implications for children’s emotional development. Developmental Psychology.

[B65-behavsci-15-00419] Russell G., Rodgers L. R., Ford T. (2013). The strengths and difficulties questionnaire as a predictor of parent-reported diagnosis of autism spectrum disorder and attention deficit hyperactivity disorder. PLoS ONE.

[B66-behavsci-15-00419] Salayev K. A., Sanne B. (2017). The strengths and difficulties questionnaire (SDQ) in autism spectrum disorders. International Journal on Disability and Human Development.

[B67-behavsci-15-00419] Schalamon J., Ainoedhofer H., Singer G., Petnehazy T., Mayr J., Kiss K., Höllwarth M. E. (2006). Analysis of dog bites in children who are younger than 17 years. Pediatrics.

[B68-behavsci-15-00419] Schohl K. A., Van Hecke A. V., Carson A. M., Dolan B., Karst J., Stevens S. (2014). A replication and extension of the PEERS intervention: Examining effects on social skills and social anxiety in adolescents with autism spectrum disorders. Journal of Autism and Developmental Disorders.

[B69-behavsci-15-00419] Scoresby K. J., Strand E. B., Ng Z., Brown K. C., Stilz C. R., Strobel K., Barroso C. S., Souza M. (2021). Pet ownership and quality of life: A systematic review of the literature. Veterinary Sciences.

[B70-behavsci-15-00419] Shalev I., Warrier V., Greenberg D. M., Smith P., Allison C., Baron-Cohen S., Eran A., Uzefovsky F. (2022). Reexamining empathy in autism: Empathic disequilibrium as a novel predictor of autism diagnosis and autistic traits. Autism Research.

[B71-behavsci-15-00419] Shoesmith E., Shahab L., Kale D., Mills D. S., Reeve C., Toner P., Santos de Assis L., Ratschen E. (2021). The influence of human–animal interactions on mental and physical health during the first COVID-19 lockdown phase in the UK: A qualitative exploration. International Journal of Environmental Research and Public Health.

[B72-behavsci-15-00419] Smees R., Rinaldi L. J., Simmons D. R., Simner J. (2023). The parent-completed glasgow sensory questionnaire: Exploring children’s sensory sensitivities and their relationship to well-being. Journal of Child and Family Studies.

[B73-behavsci-15-00419] Sönmez D., Jordan T. R. (2024). Investigating associations between cognitive empathy, affective empathy and anxiety in adolescents with autism spectrum disorder. International Journal of Developmental Disabilities.

[B74-behavsci-15-00419] Spain D., Sin J., Harwood L., Mendez M. A., Happé F. (2017). Cognitive behaviour therapy for social anxiety in autism spectrum disorder: A systematic review. Advances in Autism.

[B75-behavsci-15-00419] Vilas S. P., Reniers R. L., Ludlow A. K. (2021). An investigation of behavioural and self-reported cognitive empathy deficits in adolescents with autism spectrum disorders and adolescents with behavioural difficulties. Frontiers in Psychiatry.

[B76-behavsci-15-00419] Wang X., Auyeung B., Pan N., Lin L. Z., Chen Q., Chen J. J., Liu S. Y., Dai M. X., Gong J. H., Li X. H., Jing J. (2022). Empathy, theory of mind, and prosocial behaviors in autistic children. Frontiers in Psychiatry.

[B77-behavsci-15-00419] Wauthier L. M., Farnfield S., Williams J. M. (2022). The role of attachment in children’s relationships with pets: From pet care to animal harm. Human-Animal Interactions.

[B78-behavsci-15-00419] World Health Organization (2023). Autism key facts.

[B79-behavsci-15-00419] Young J., Pritchard R., Nottle C., Banwell H. (2020). Pets, touch, and COVID-19: Health benefits from non-human touch through times of stress. Journal of Behavioral Economics Policy.

[B80-behavsci-15-00419] Yu C. (2024). A systematic review of the positive effects of pets on child development. Communications in Humanities Research.

